# Mapping the susceptibility of reefs to rubble accumulation across the Great Barrier Reef

**DOI:** 10.1007/s10661-024-12344-4

**Published:** 2024-01-29

**Authors:** Shu Kiu Leung, Peter J. Mumby

**Affiliations:** Marine Spatial Ecology Lab, School of the Environment, University of Queensland, Level 5, Goddard Building, St. Lucia, QLD, Brisbane, 4072 Australia

**Keywords:** Coral rubble, Coral restoration, Rubble accumulation, Reef recovery

## Abstract

Disturbance-induced rubble accumulations are described as “killing fields” on coral reefs as coral recruits suffer high post-settlement mortality, creating a bottleneck for reef recovery. The increasing frequency of coral bleaching events, that can generate rubble once coral dies, has heightened concerns that rubble beds will become more widespread and persistent. But we currently lack the tools to predict where rubble is most likely to accumulate. Here, we developed a modelling framework to identify areas that are likely to accumulate rubble on forereef slopes across the Great Barrier Reef. The algorithm uses new high-resolution bathymetric and geomorphic datasets from satellite remote sensing. We found that 47 km of reef slope (3% of the entire reef surveyed), primarily in the southern region, could potentially reach 50% rubble cover. Despite being statistically significant (*p* < 0.001), the effects of depth and aspect on rubble cover were minimal, with a 0.2% difference in rubble cover between deeper and shallower regions, as well as a maximum difference of 0.8% among slopes facing various directions. Therefore, we conclude that the effects of depth and aspect were insufficient to influence ecological processes such as larval recruitment and recovery in different coral communities. Maps of potential rubble accumulation can be used to prioritise surveys and potential restoration, particularly after major disturbances have occurred.

**Supplementary Information:**

The online version contains supplementary material available at 10.1007/s10661-024-12344-4.

## Introduction

**Fig. 1:**
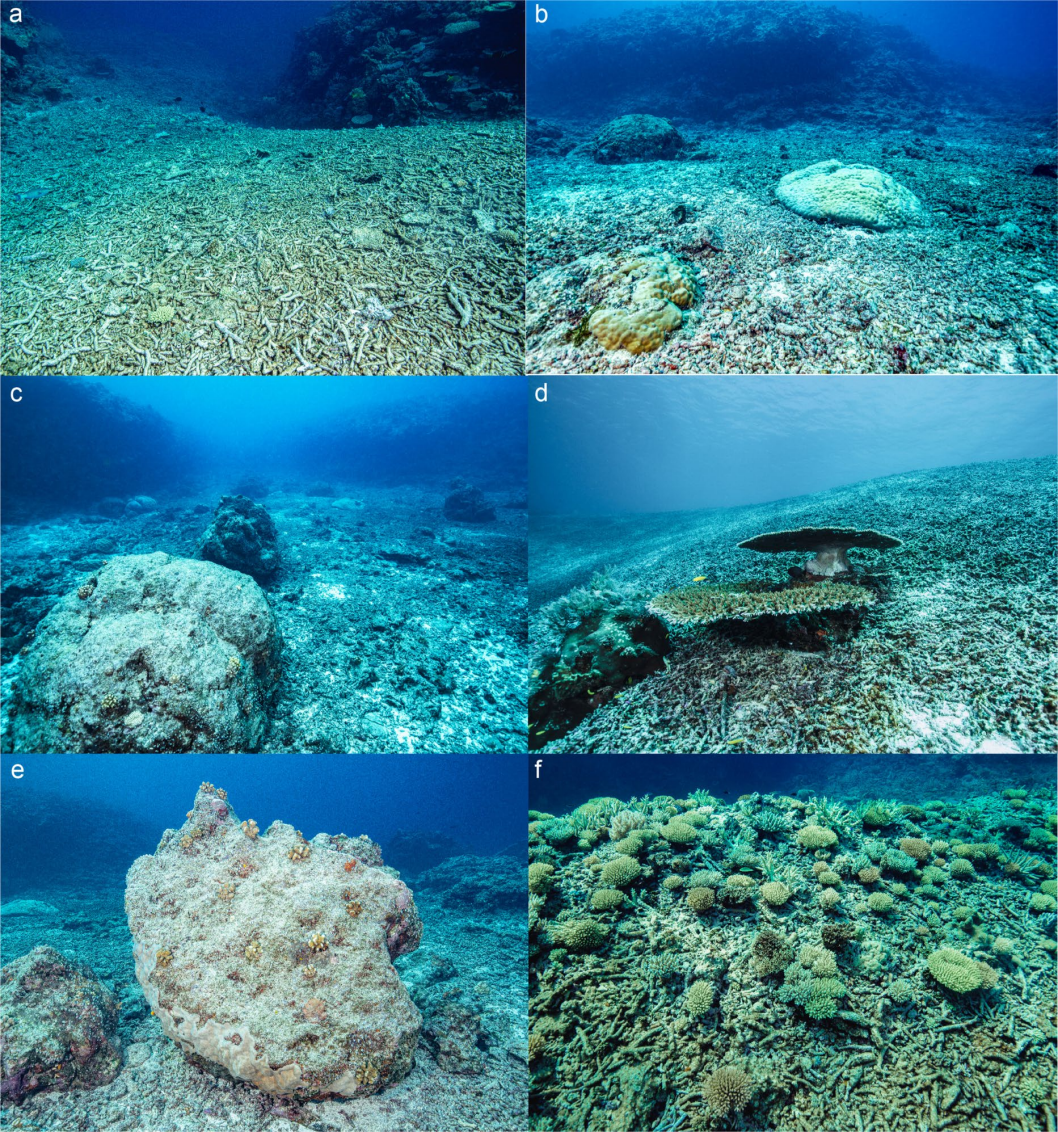
Features of rubble on coral reefs showing newly-formed beds after disturbance (**a**), filling of gullies to create a flat, homogenous substrate (**b**, **c**), the occasional existence of corals that were spared from disturbance (**d**), the initial confinement of coral recovery to elevated non-rubble habitats (**e**) and finally the recruitment of coral cohorts once the rubble has stabilised (**f**)

Coral reefs worldwide are subject to increasingly frequent and severe damage from disturbances, including coral bleaching, cyclones and anthropogenic activities, often creating large amounts of rubble (Done, 1992; Fabricius, & De’Ath, G., Puotinen, M. L., Done, T., Cooper, T. F.,, & Burgess, S. C., 2008; Fox et al., 2003; Harmelin-Vivien, 1994; Riegl, 2001). The term “rubble” refers to fragments of coral skeleton or reef rock derived from mechanical or chemical abrasion that have distinct morphology (branching, plate and boulder) and size ranges (from > 2 mm to > 1 m) depending on the assemblage composition of the reef and the characteristics of disturbances (Rasser & Riegl, 2002; Wolfe et al., 2021; Woodley et al., 1981) (Fig. 1). In addition to direct damage, indirect effects such as weakened coral skeleton and increased fragmentation after mortality can also contribute to rubble generation (Ceccarelli et al., 2020). Morais et al. (2022) expand on this process, highlighting the rapid erosion of dead coral colonies, particularly the complete disintegration of colonies with complex growth forms within 60 months, which may play a significant role in rubble formation rather than reef growth. Rubble formation is considered a part of the natural destruction and regeneration cycles that shape the diversity of the reef (Rogers, 1993). However, as climate change intensifies disturbances (McWhorter et al., 2022), rubble formation is expected to accelerate and increase the prevalence of rubble on reefs (Cheal et al., 2017; Hoegh-Guldberg et al., 2007).

Persistent rubble fields can reduce reef structural complexity and inhibit reef recovery, raising concerns that future disturbance rates may surpass recovery rates (Ceccarelli et al., 2020; Fox et al., 2003; Knutson et al., 2020; Ortiz et al., 2018; Viehman et al., 2018). The “flattening” of reef structure has been documented in different regions as reef ecosystems degrade (Alvarez-Filip et al., 2009; Elliott et al., 2018). The loss of structural complexity can lead to significant ecological and socio-economic impacts, including shifts in coral reef functional processes, as well as a reduction in species diversity, fisheries productivity and coastal protection offered by the reef, thus affecting the livelihoods of millions of people (Graham et al., 2006; Harris et al., 2018; Morais et al., 2020; Rogers et al., 2014). In addition to flattening the reef, rubble fields can also impede recovery processes (Kenyon et al., 2020; Rasser & Riegl, 2002; Woodley et al., 1981). Coral recovery in rubble fields is largely determined by the stability of the substrate, which is essential for the survival of coral larvae (Yadav et al., 2016). Stabilised rubble fields are favourable for coral settlement owing to the abundance of cryptic microhabitats, which help reduce predation and competition for space with macroalgae (Biggs, 2013; Doropoulos et al., 2016; Edmunds et al., 2004). Coral recolonisation can take place in as little as 10 months when the rubble field is consolidated through binding processes mediated by sponges and encrusting taxa (Wulff, 1984). However, the stabilisation process can be interrupted in high-energy environments that promote rubble movement, leading to mechanical abrasion and smothering of newly settled coral recruits, as well as causing damage to adjacent coral colonies (Brown & Dunne, 1988; Clark & Edwards, 1995; Viehman et al., 2018). A case study on the upper Florida Keys shelf observed a 52% loss of juveniles in a rubble field, as opposed to 10–40% at a nearby consolidated reef site, despite having similar densities of coral recruits and species composition (Cameron et al., 2016). Viehman et al. (2018) found that continued mobilisation of rubble in high-energy environments can result in a positive feedback loop in which rubble becomes smaller due to frequent erosion and mobilises at lower levels of hydrodynamic forcing. Thus, coral recovery on persistent rubble is therefore unlikely and may take decades even in the absence of further disturbances (Dollar & Tribble, 1993; Riegl, 2001). Indeed, several studies have documented little to no recovery in rubble-dominated sites years after previous disturbances (Chong-Seng et al., 2014; Fox & Caldwell, 2006; Victor, 2008). As a result, there is growing concern that future disturbance rates may exceed the capacity of coral reefs to recover, particularly because recovery rates from rubble are likely slower than those on firm structure, thereby requiring even longer recovery windows (Chong-Seng et al., 2014; Fox et al., 2019; Raymundo et al., 2007; Yadav et al., 2016).

The mechanisms of rubble accumulation on reef slopes, where rubble is likely to become a persistent bottleneck for reef recovery, are heavily influenced by the area’s bathymetric properties including its slope and profile (Dollar & Tribble, 1993; Harmelin-Vivien & Laboute, 1986; Highsmith et al., 1980; Rasser & Riegl, 2002; Shannon et al., 2013; Thornborough, 2012). Although the majority of disturbance-generated rubble accumulates as talus at the foot of forereef slopes, some may stay as gently sloping ramparts or remain in depressions, potentially interfering with reef recovery (Scoffin, 1993). A study in Hawaii found that rubble fragments transported and deposited on the reef slope remain unconsolidated after 20 years (Dollar & Tribble, 1993). Rubble can also accumulate and cover large areas on reef flats but will often undergo erosion into smaller pieces and be transported shoreward to form sand cays and reef islands in lagoons (Hughes, 1999; Thornborough & Davies, 2011). On steeper reef slopes (> 45°), “avalanches,” which refers to the offshore and downslope movement of rubble, can take place and destroy reef communities in their path (Harmelin-Vivien & Laboute, 1986; Scoffin, 1993). However, on gentler parts of the slope, rubble will likely accumulate or be transported shoreward depending on the hydrodynamic properties of the environment (Hughes, 1999). The profile of the reef slope dictates where rubble accumulates as rubble preferentially deposits in groove-like structures, which can act as channels to facilitate wave propagation and allow rubble transportation onto these specific zones (Shannon et al., 2013). Hydrodynamic properties and disturbance regimes, despite also influencing the distribution of rubble fields at a smaller scale, are more strongly tied to rubble formation and stabilisation processes than rubble accumulation (Harris & Vila-Concejo, 2013; Rasser & Riegl, 2002; Viehman et al., 2018). Hence, slope and profile are the key drivers in rubble accumulation, and a detailed spatial analysis should help identify vulnerable areas on a reef. Based on these key drivers, this study assumes that relatively flat and groove-like locations are vulnerable to rubble accumulation which may then impair reef recovery. Whether accumulated rubble is able to stabilise will depend on several factors, including agitation from wave-related energy, and is beyond the scope of this study. Our key focus is to identify areas where rubble might collect.

Here, we present a simple modelling approach to identify reefs that are likely to be susceptible to rubble accumulation once large areas of coral have died and eroded. The method uses newly available bathymetric and geomorphic datasets across mid- and outer-shelf reefs of the Great Barrier Reef (GBR), which have sufficient water clarity for optical remote sensing. By analysing reef bathymetric profiles to extract rubble-prone areas, this study aims to (1) estimate the extent of reef slope that is susceptible to rubble accumulation; (2) identify reefs that are at the highest risk of rubble accumulation as well as their spatial distribution and (3) examine the effects of aspect and depth on rubble accumulation. We point out upfront that our mechanistic understanding of rubble retention is not sufficiently well developed to estimate the absolute amount of rubble accumulation. Thus, our goal is to estimate the relative vulnerability of reefs to rubble accumulation based on morphological features. We hypothesise that the potential rubble cover will vary with depth and aspect as hydrodynamic regimes largely shape reef geomorphology (Hopley et al., 2007; Montaggioni & Braithwaite, 2009).

## Materials and methods

### Overview

**Fig. 2:**
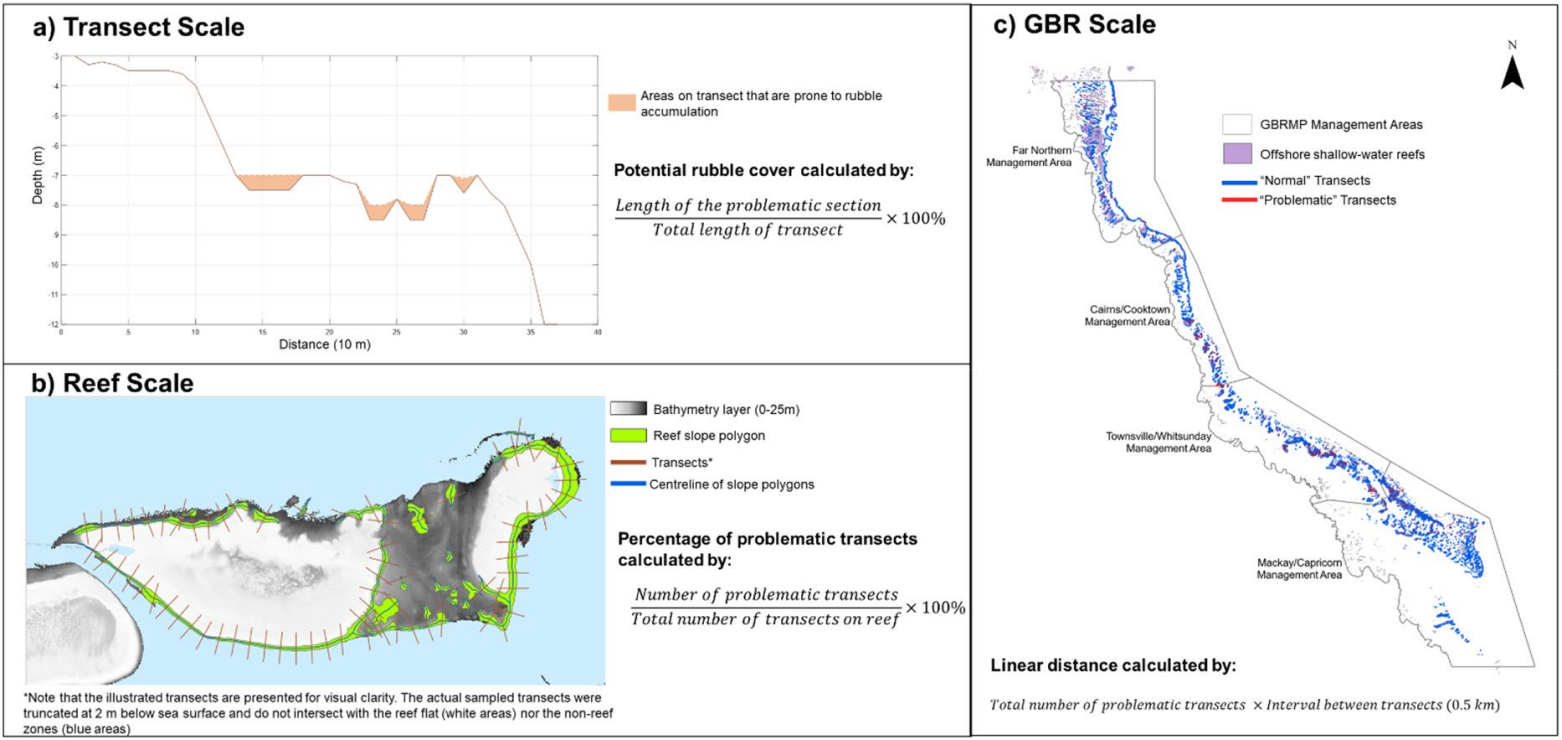
Diagram demonstrating the calculation of different metrics at a transect scale (**a**), reef scale (**b**) and a GBR scale (**c**). **a** shows the profile of a sample transect along with areas where rubble may accumulate. **b** uses Heron reef as an example to illustrate how these transects are placed parallel to the reef slopes. **c** shows an example distribution of problematic transects across the GBR. Here, problematic transects are those where the rubble cover goes beyond a defined threshold

The model was developed in ESRI ArcGIS Pro (2.8.0), MATLAB R2021b (9.11), and R (4.2.1) to characterise the susceptibility of coral reefs to rubble accumulation (Fig. S1). A one-dimensional algorithm was developed to evaluate the risk of rubble accumulation, expressed as the potential rubble cover, at a transect level. Bathymetric profiles were extracted and analysed from transects sampled throughout the Great Barrier Reef Marine Park (GBRMP) to determine the extent of rubble accumulation. Each transect was orientated parallel to the reef slope, extending from the shallows towards deeper areas (Fig. 2). We then calculated reef-scale metrics, including the percentage of transects in which rubble was predicted to exceed defined thresholds. To create a GBR-scale metric, we examined the number of transects meeting a particular rubble criterion, and expressed their number as the linear distance, in kilometres, along the reef axis (i.e. perpendicular to the transects and at constant depth). Given that several of the algorithm’s components are uncertain, we explored the sensitivity of our conclusions to model assumptions.

### Site description and sources of data

The study area is approximately 344,000 km^2^, spanning 14 degrees of latitude from 10°20′S in the north to 24°30′S in the south (Brodie & Waterhouse, 2012). The forereef slopes of 1834 offshore shallow water reefs were sampled across the GBRMP for modelling rubble accumulation. Nearshore reefs were excluded due to the limitations in their visibility and the coverage of the available data sources. Following the removal of data inconsistencies (see Transect sampling), 1706 reefs were ultimately chosen for susceptibility mapping. Reef slopes, both exposed and sheltered, were isolated from individual reefs and selected as areas of interest due to their higher potential for rubble accumulation (Dollar & Tribble, 1993; Highsmith et al., 1980; Rasser & Riegl, 2002).

Publicly available, open-source data layers depicting the reef boundaries, bathymetry and geomorphic zonation in the GBRMP (Table 1) were analysed using ArcGIS Pro Version 2.8.0. The indicative boundaries of reefs in GBRMP were extracted from the “GBR Features” polygon layer acquired from GBRMPA Geoportal (Great Barrier Reef Marine Park Authority, 2020). Islands, cays, rocks and dry reefs were excluded from the layer during the extraction. The layer was used to group results by reef boundaries in statistical analyses and data visualisation. The bathymetry data layer was derived from optical Sentinel-2 satellite imagery at 10-m resolution, with a vertical accuracy of ± 1 m and a maximum depth of 25 m corrected to mean sea level (Hedley et al., 2018; Roelfsema et al., 2021). The source bathymetry data were divided into four parts based on management areas (Great Barrier Reef Marine Park Authority, 2021b). The four parts were mosaicked and collated into a common geographic coordinate system to ensure data integrity and continuity during transect sampling and rubble modelling. The geomorphic zonation layer was mapped by the Remote Sensing Research Centre using a machine learning approach which combines satellite imagery, environmental attributes and occurrences of geomorphic zonation (Roelfsema et al., 2021). According to Roelfsema et al. (2021), the data were mapped at 10-m resolution for depths ranging from 0 to 20 m (corrected to mean sea level) and has an overall accuracy of 68%. The layer was used to identify and isolate the reef slopes in preparation for transect sampling.

**Table 1:** Data sources for the spatial analysis

Data layer name	Description	Source
GBR Reef	Vector layer showing indicative boundaries of shallow water reefs in GBRMP.	Great Barrier Reef Marine Park Authority (2020)
GBR10 Bathymetry	Raster layer combining four parts of the bathymetry data of different management areas and projected onto the same geographic coordinate system, WGS 1984.	Great Barrier Reef Marine Park Authority (2021b)
GBR10 Geomorphic	Raster layer showing the geomorphic zones in GBRMP.	Great Barrier Reef Marine Park Authority(2021a)

While rubble is extensively surveyed as a substrate type throughout the GBRMP, rubble cover is often underreported, and there are no baseline data for rubble parameters, such as size, volume and density of accumulations (Ceccarelli et al., 2020). Most of the literature focuses on rubble formation while the consequences of rubble accumulation remain relatively understudied (Kenyon et al., 2020; Rasser & Riegl, 2002; Thornborough, 2012).

### Transect sampling

Transects (*n* = 32,603) were generated along reef slopes at a separation distance of 500 m and aligned perpendicular to the reef axis to capture the variations in depth down the slope. Reef slopes isolated from the GBR10 Geomorphic raster layer were converted into polygons (Conversion toolbox) and simplified (Cartography toolbox) to remove potential errors during transect generation. The conversion process was necessary for the calculation of the centrelines for generating transects. To reduce data noise while maintaining data integrity, all polygons smaller than 0.01 km^2^ were removed after the polygons were simplified using the “retain critical bends” algorithm with a simplification tolerance of 500 m. The simplification process did not alter the values of the bathymetry layer but eliminated inconsistencies along the boundaries of the reef slopes. Centrelines were generated using the Topographic Production toolbox within the polygons to indicate the line along which perpendicular transects can be created. Some centrelines may not exactly reflect the alignment of the slope when the reef is irregularly shaped. These centrelines were manually removed to minimise inconsistencies during transect generation. Transects with a length of 500 m were generated along the centrelines using the Data Management toolbox to ensure they cross the entire reef slope. Both ends of the transects were truncated at a depth limit of − 2 m to avoid sampling reef crests and reef flats (where the minus sign indicates below sea surface). The depth limit was selected based on the mean depth values for reef crests (M = − 1.32 m, SD = 0.5 m) and outer reef flats (M = − 1.49 m, SD = 0.87 m). The truncated transects were grouped by individual reefs on the GBR reef layer using Spatial Join in the Analysis toolbox. Transects were allocated to their closest reefs within a 1000-m searching distance radius. This step ensures that all transects were allocated correctly and accounts for any discrepancies between the GBR10 Bathymetry and GBR Reef layers. The average length of generated transects was 287.2 m (*SD* = 173.8).

To account for transects facing different directions, their mean aspect was calculated using a series of ArcGIS Pro tools, with reference to the python script tool by Beyerhelm (2013). The Aspect tool (Spatial Analyst) was used to generate the aspect from each cell of the bathymetry layer. Using the python script tool, a buffer of 10 m was created around each transect to calculate the zonal mean aspect. The following formula shows the fundamental calculation in the python script tool:

$$\left[360+\textrm{atan}2\left(\textrm{s},\textrm{c}\right)\left(\frac{180}{\uppi}\right)\right]\operatorname{mod}360$$

where **s** and **c** are the zonal mean values of the sine and cosine of the aspect, respectively. **atan2** represents the two-argument arctangent function. The result is an angle in unit degrees that represents the average aspect of each transect and was classified into the four directions of North, East, South and West based on its value.

Depth values were extracted from the transects and compiled into a data table for further analysis in the rubble accumulation algorithm. The extraction of bathymetric profiles required the Stack Profile tool in the 3D Analyst toolbox, which allows bilinear interpolation of depth values on the GBR Bathymetry layer for the grouped transects. The output of the tool was a table denoting the bathymetric profiles of the transects, with depth values recorded at each point on the transect spaced by 10 m. The output was later cleaned and prepared in R version 4.2.1 for further analysis. Duplicated records and missing values were also removed to maintain data consistency. After data cleaning, 22,562 transects which accounted for 1706 reefs were left for subsequent analyses.

### Rubble accumulation algorithm

**Fig. 3:**
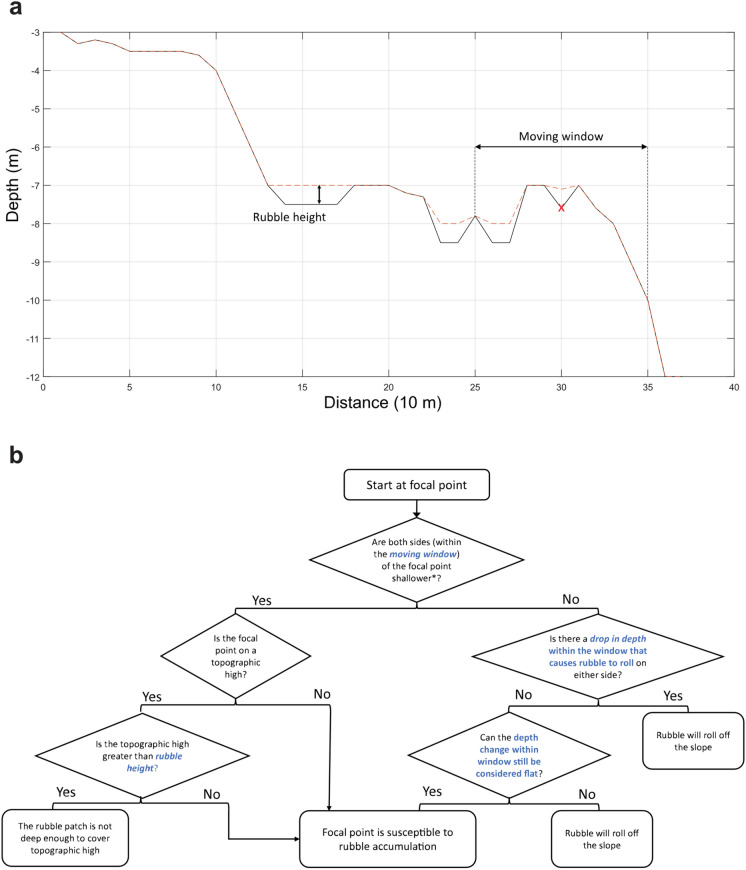
Diagram showing an example graphical output of a transect using the rubble accumulation model (**a**) and the decision tree to demonstrate the logic in the rubble accumulation model. (**b**) The base case parameter values were used in **a** to compute the results. The solid line represents the original transect profile, whereas the dotted line represents the new transect profile after filling susceptible areas with rubble with the value of *rubble height*. The red “x” mark represents an example of a focal point to demonstrate its relationship with the *moving window*. In **b**, the term shallower indicates that the focal point is in a depression when both sides have a smaller depth value compared to the sum of the depth of the focal point and *rubble height.* **b** also shows the decision-making process involving the parameter *Depth range within window that causes rubble to roll* and *Depth change within window that is still considered flat*

The algorithm was designed to locate rubble-prone areas on transects and calculate the percentage of potential rubble cover in different depth regions. Sections of transects were classified as either “shallow” or “deep”, based on their depths. “Shallow” sections are less than 10 m in depth; otherwise, they are considered “deep”. The algorithm scans and evaluates each transect point to determine whether rubble is likely to accumulate, based on four input parameters—*Moving window*, *Rubble height*, *Depth range within window that causes rubble to roll* and *Depth change within window that is still considered flat* (Fig. 3). A detailed description of each model parameter is given in Table 2. A base case of parameter values was determined using the distribution of transect lengths and critical slopes, in addition to expert opinion. The base case represents the most realistic scenario and acts as a reference point for the sensitivity analysis.

**Table 2:** Input parameters for the rubble accumulation model

Parameter name	Description	Base case value	Unit
Moving window	Spatial scale over which reef slope is evaluated around a focal point. Helps determine whether rubble will naturally roll down to a deeper location	5 pixels = 50 m	Metres
Rubble height	Minimum thickness of accumulated rubble that causes problems for coral recovery. Indentations on the reef surface that are shallower than this may accumulate rubble but are not considered problematic	0.5	Metres
Depth range within window that causes rubble to roll	The magnitude of change in depth within the moving window that decides if the point is on or close to a steep slope where rubble may roll off. Rubble cannot accumulate on the focal point if the depth change within the moving window is greater than this	5	Metres
Depth change within window that is still considered flat	The maximum permitted value of the change in depth within the moving window that would allow the rubble to remain on the focal point	0.5	Metres

The ability of rubble to accumulate is determined within a floating horizontal window that passes along the entirety of each transect and stops at every 10-m interval (“focal point”) to check for possible rubble accumulation. The algorithm evaluates if each focal point will accumulate rubble depending on the topographical features within the floating horizontal window, which is determined by the value of the parameter *Moving window*. The value of *Moving window* is typically set at 5 pixels (50 m) (Table 2), meaning that the floating window extends 50 m to both sides of the focal point. If a transect is 500 m long, the window will shift its position 50 times, moving with the focal point until it reaches the end of the transect. Rubble will accumulate in local depressions that exceed a depth of 0.5 m and on “flat” sections of reef, defined as having a variation in depth of ≤0.5m. There are some exceptions to this. For example, rubble cannot accumulate on a local topographic high or if the “flat area” is proximal to a steep slope, where rubble is likely to roll away. Proximity and slope are defined by the variation in depth ≥ 5 m within the evaluation window (Table 2). The parameterisation is somewhat subjective and based on two considerations. First, personal observations of one of the authors (PJM) studying the accumulation of rubble on reefs after cyclone impacts in Belize (Mumby, 1999), Moorea (Mumby et al., 2016) and Palau (Roff et al., 2015). In several cases, rubble accumulated for at least a decade, yet it rolled off reef structures that exceeded 5 m in height from surrounding areas. Second, the use of satellite-derived bathymetry, while being the only means of obtaining continuous data across the GBR, does have limited sensitivity. Despite the relatively high radiometric resolution of the Sentinel II imagery, bathymetry algorithms cannot resolve small-scale changes in depth, such as 0.1 m (Green et al., 2000). Therefore, we felt it appropriate to specify minimal depth differences of 0.5 m, which are resolvable in the data. We note that rubble accumulations of < 0.5 m are able to inhibit coral recruitment (Mumby, pers. obs.), but such levels could not be specified accurately in a GBR-wide algorithm.

### Calculating reef susceptibility

We defined susceptibility as the percentage of transects per reef having a potential rubble cover higher than a given threshold. This approach highlights problematic areas within reefs that have a high potential of rubble accumulation. Since there is limited literature on the critical value of rubble cover at which the recovery of the reef would be impaired, we chose values in the range observed in the field, following Typhoon Bopha in Palau, where accumulated rubble represented up to 50% of the reef. We therefore used thresholds of 30%, 40% and 50% cover (though a full range of 10 to 90% are shown for some results). The distribution of percentages was plotted using the “ggpubr” package in R to determine suitable thresholds for visualising the spatial variability of susceptibility to rubble in ArcGIS Pro (Kassambara, 2020). The number of transects exceeding the thresholds was multiplied by the interval between transects to estimate the extent of reefs (in kilometres) that are thought to be susceptible. For example, if there were ten susceptible transects in a reef and the transects were placed 500 m apart, the linear distance would be 5 km.

A depth-stratified analysis was performed to compare the susceptibility of reef slopes facing various directions in different depth regions using a beta regression model in R 4.2.1. The R packages “glmmTMB”, “DHARMa” and “betareg” were used for the analysis (Grün et al., 2012; Hartig, 2022). A beta regression model was used to test the fixed effects of depth and aspect on the proportion of potential rubble cover of the transects. The proportion data were linearly transformed and compressed to avoid zeros and ones using the methods discussed by Smithson and Verkuilen (2006). A two-component mixture model was used to fit the data due to the bimodal distribution of residuals in the data when fitting a generalised linear mixed model. The mixture model assumes that the data can split into two groups that fit two different beta distributions to reflects its bimodality.

### Sensitivity analysis

The one-at-a-time method was utilised to assess the sensitivity of the rubble accumulation algorithm to the four input parameters (Hamby, 1994). Parameters were changed by ± 20% relative to the base case in each run, yielding three scenarios for each parameter. The analysis evaluated two output variables: the susceptible reef count and the reef’s susceptibility ranking. The sensitivity of the susceptible reef count was expressed as the mean percentage change in the number of reefs containing transects exceeding the critical thresholds relative to the change in input parameter values. For the sensitivity of reef ranking, the top 10% of highly susceptible reefs were chosen and ranked based on their percentage of transects with potential rubble cover over critical thresholds. The greater the percentage, the higher the ranking of the reef. This variable assesses the algorithm’s consistency in the selection of highly susceptible reefs when parameter values are varied. The absolute change in reef ranking of the top 10% of reefs against the ± 20% change in parameter values was averaged for every threshold category to compute the overall sensitivity of the model’s decision. The parameters were then ranked based on sensitivity analysis results to determine which parameters contribute the most to the model’s variability.

## Results

### Linear distances and distributions of rubble accumulation

**Fig. 4:**
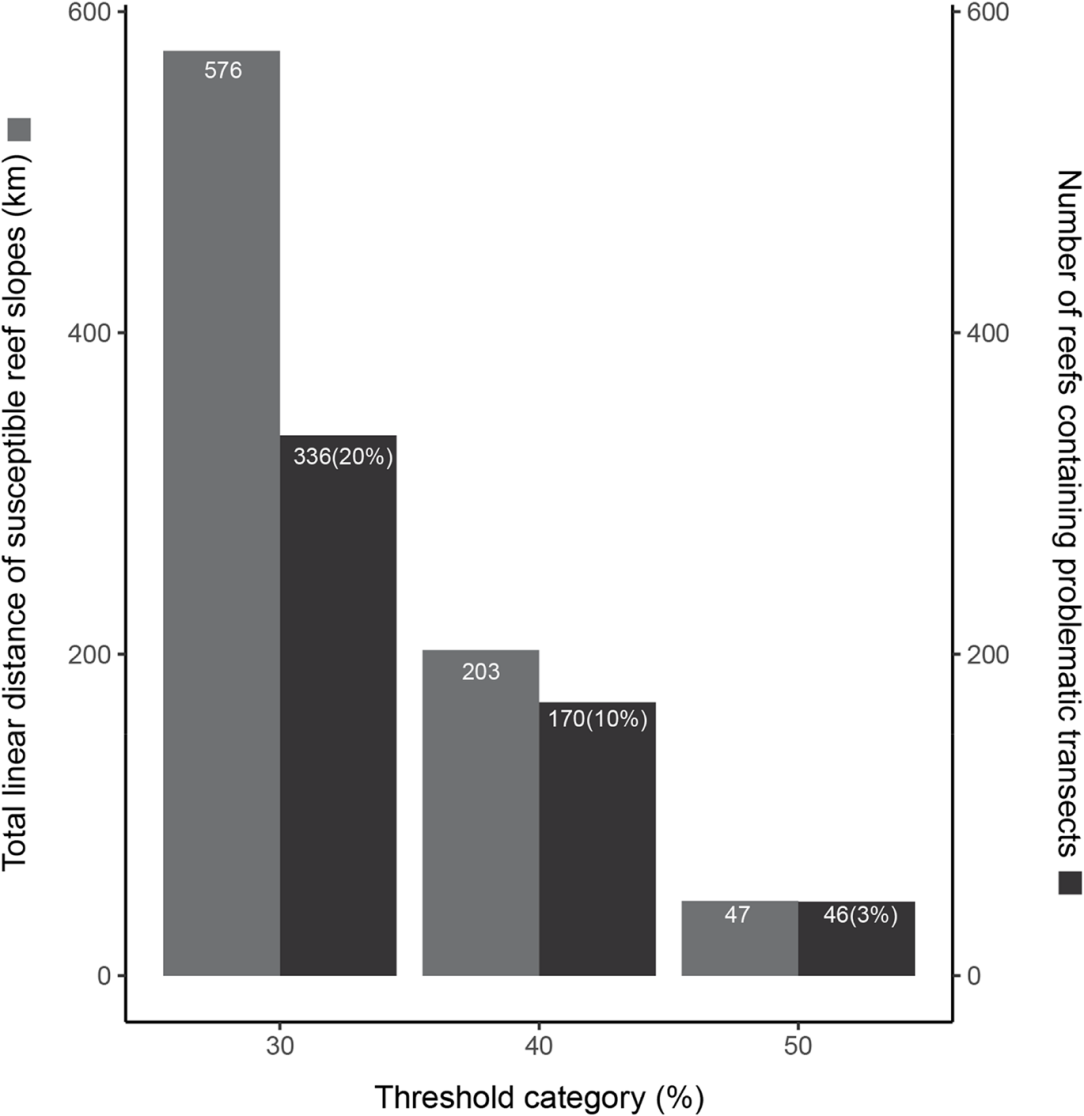
Summary of rubble accumulation parameters across critical threshold categories, including the total linear distance of susceptible reef slopes across the GBR (km), the number of reefs containing problematic transects and the percentage of reefs containing problematic transects. The term “problematic transects” indicates transects with potential rubble cover exceeding the threshold

**Fig. 5:**
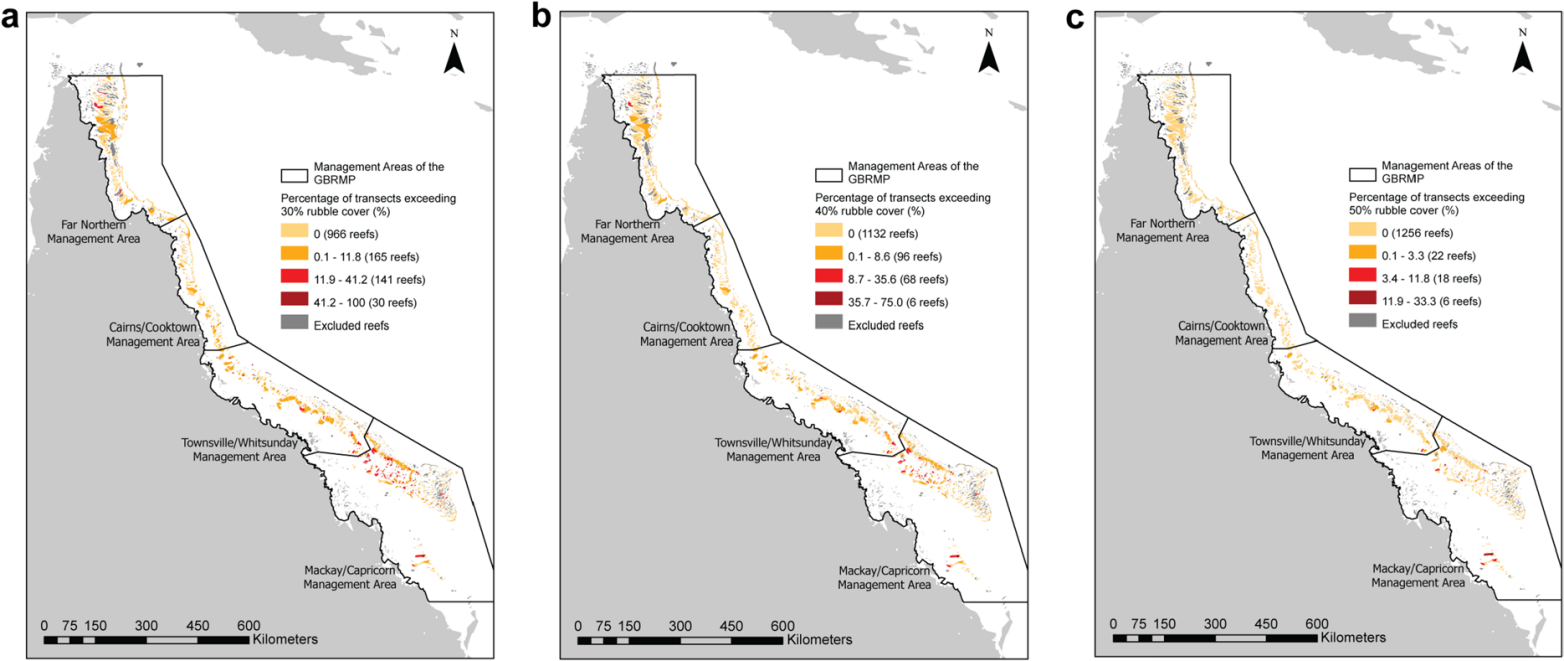
Spatial distribution of reefs with proportion of transects exceeding critical rubble cover thresholds of **a** 30%, **b** 40% and **c** 50%. The number in brackets next to the percentages indicates the number of reefs that fall under the category. 404 reefs with zero potential rubble cover across the entire extent are marked as “Excluded reefs” in grey. Reefs in the 0% category are capable of accumulating rubble but their cover does not exceed the critical thresholds 30%, 40% and 50%

Approximately one-quarter of reefs (404/1706, 23.7%) was predicted to have no rubble accumulation potential. As the threshold of rubble accumulation per transect was increased from 30 to 50%, there was a sharp reduction in the number of reefs affected and the total length of problematic reef (Fig. 4). For example, if we consider that ≥ 30% of a forereef profile (transect) gathering rubble is problematic, then 336 reefs contain problematic transects. If we set a higher threshold of 50% rubble cover, then the number of reefs drops to 46 (Fig. 4), and their spatial distribution is more limited. The latitudinal range of susceptible reef under the 30% rubble cover threshold category spans 13 degrees, from 10°40′S to 23°40′S. In the case of the 50% threshold, the range is more restricted, covering only 6 degrees from 17°10′S to 23°40′S, with the exception of one outlier reef located at 11°30′S. Reefs with the higher percentages of problematic transects mostly occurred in the central to the southern GBR, including the Townsville/Whitsunday and Mackay/Capricorn management area, regardless of the threshold category (Fig. 5).

**Fig. 6:**
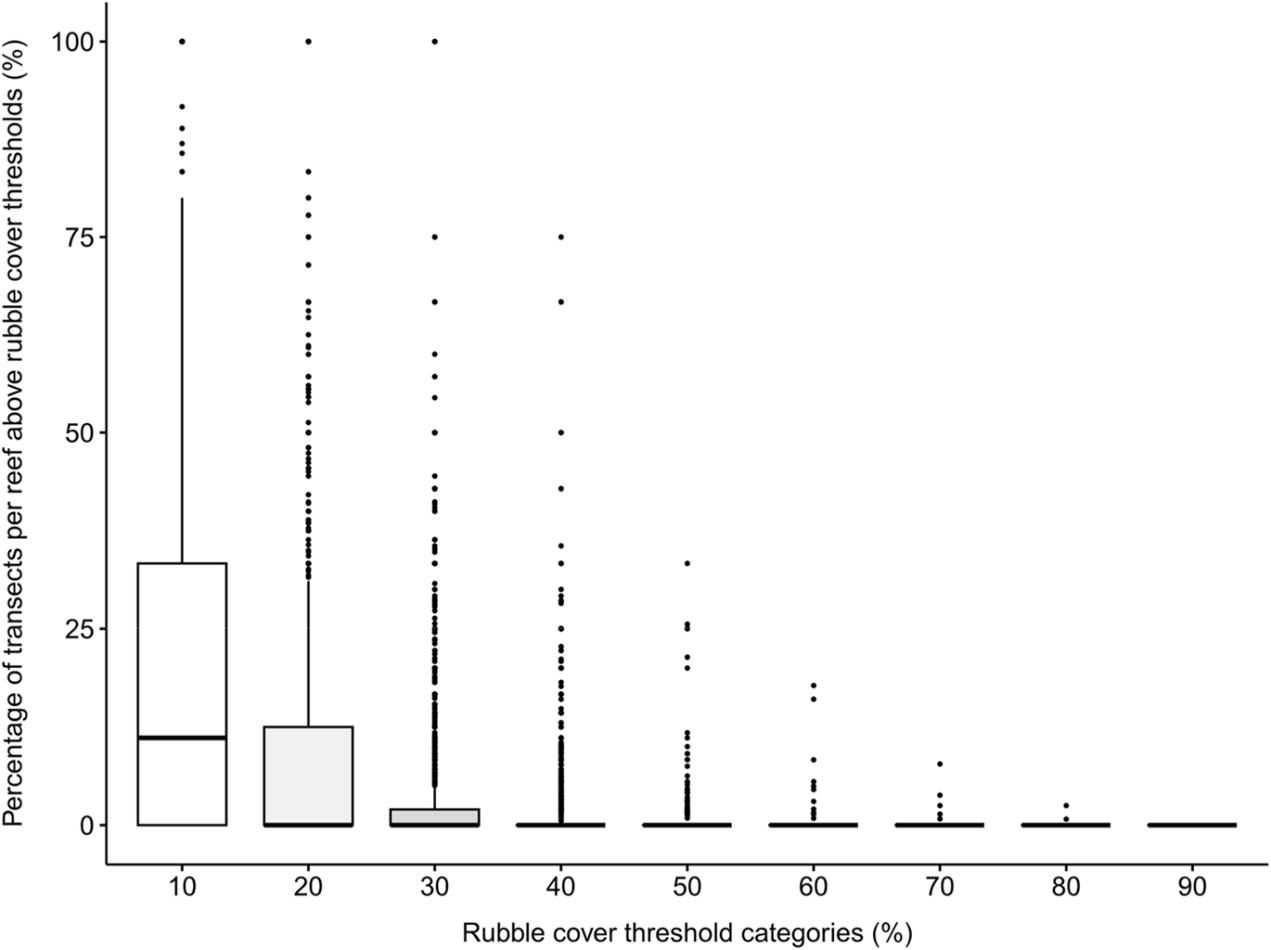
Boxplot showing the distribution of the percentage of transects per reef exceeding rubble cover threshold categories of 10%, 20%, 30%, 40%, 50%, 60%, 70%, 80% and 90%. Reefs with zero potential rubble cover were excluded

**Fig. 7:**
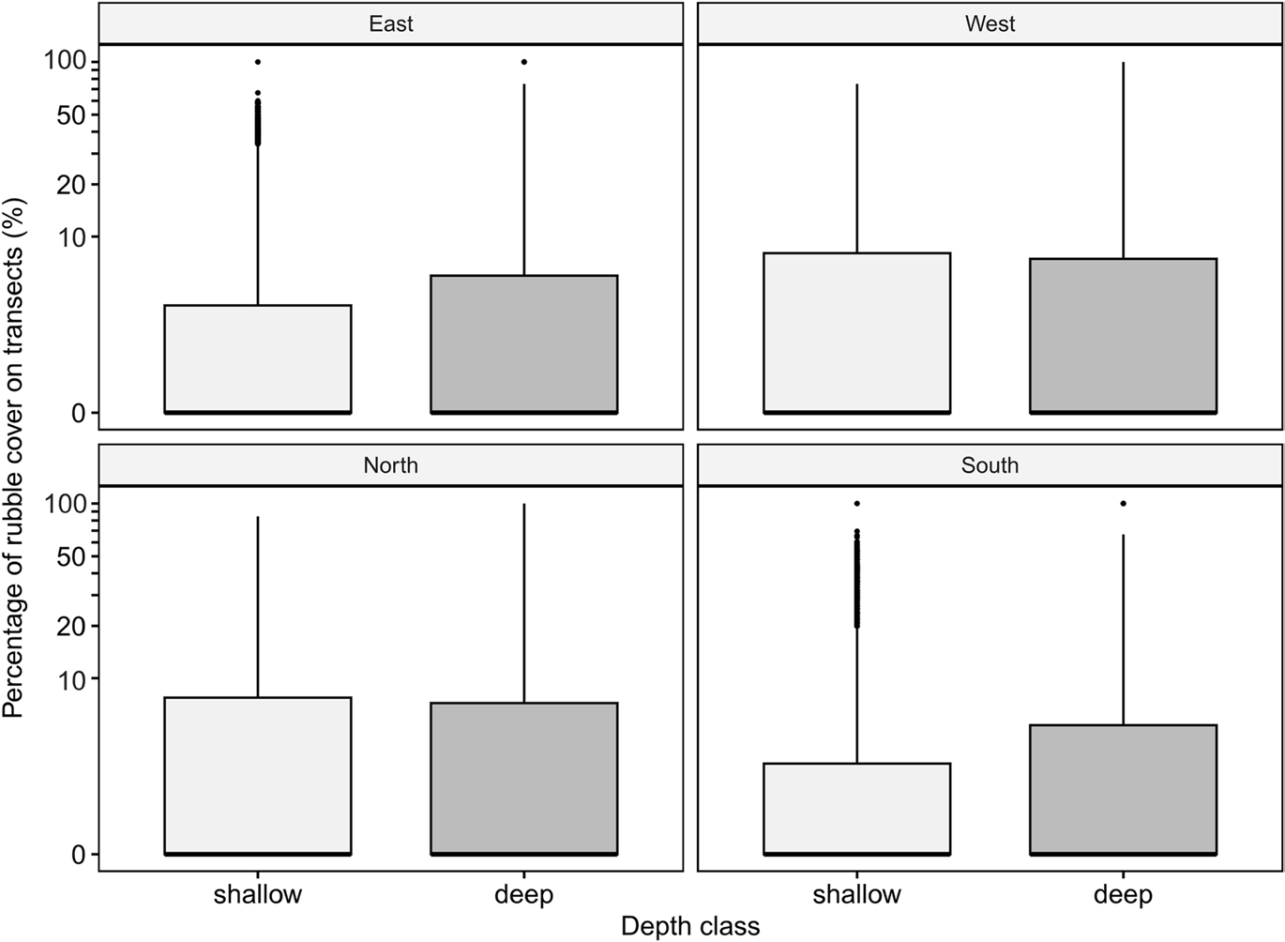
Boxplot comparing the distribution of the percentage of rubble cover on transects in shallow (depth ≤ 10 m) and deep regions (depth > 10 m), stratified by four aspect categories: East (top left), West (top right), North (bottom left) and South (bottom right). The ticks of the y axis were placed in base 10 logarithmic scale for better visualisation of the skewed distributions

There were two notable observations regarding the positively skewed distributions of rubble accumulation data grouped by individual reefs, depth and aspect. First, few reefs have a high percentage of transects with rubble exceeding the threshold (Fig. 6). With the exception of the lowest threshold category (10%), the median percentage of transects per reef with rubble greater than the threshold is zero for all threshold categories (i.e. the proportion of the average reef with problematic rubble typically falls in the lower 50th percentile). The median percentage of transects per reef with minor rubble (10% threshold) was 11.1%, and the interquartile range was 33.3%. Secondly, when assessing potential rubble cover on transects stratified by depth and aspect, the majority of transects had a low potential rubble cover, with only a few extremities completely covered by rubble regardless of depth and aspect (Fig. 7). The distributions of percentage rubble cover on transects of various depths and aspects had a median of 0% potential rubble cover and similar ranges. All boxplots in Fig. 7 had an upper quartile value that was less than 10%, indicating that at least 75% of the transects had less than 10% potential rubble cover no matter the depth or aspect. These observations suggest that the risk of rubble accumulation is both patchy and localised, as the overall percentage of reef areas containing transects with high rubble cover is remarkably low.

### Effects of aspect and depth on rubble accumulation

The relationship between depth, aspect and the potential rubble cover was determined by the regression group to which the transect was assigned (Table 3). The regression model assigned 7703 transects to the first group while 31,919 transects were assigned to the second group based on the values of posterior probabilities. No significant interaction effect was found between the independent variables in either of the groups (*p* = 0.257 for group 1 and *p* = 0.805 for group 2). There is moderate evidence showing that deeper regions (>10 m) in the first group have a potential for rubble accumulation that is 39% higher than that of shallower regions (deep = 74 ± 16% rubble cover; shallow = 35 ± 16% rubble cover; *z* = 1.95). The level of statistical significance is marginal (*p* = 0.051). In the second group, the mean percentage cover of rubble on transects in deeper regions was significantly higher than in shallower regions by 0.2% (deep = 5.2 ± 0.051% rubble cover; shallow = 5.0 ± 0.072% rubble cover; *z* = 3.37; *p* < 0.001). In comparison to those facing north (5.0 ± 0.07% rubble cover), transects facing east or south have a significantly lower rubble potential (4.5 ± 0.062% and 4.4 ± 0.058% rubble cover; *z* = − 8.60 and − 10.55; both *p* < 0.001), while there is moderate evidence suggesting that reefs facing west have the highest risk of rubble (5.2 ± 0.075% rubble cover; *z* = 2.29; *p* = 0.022). Although statistical significance was observed in both depth and aspect results, the effect sizes are generally small in the second regression group.

**Table 3:** Significant results of the 2-component beta regression mixture model for each variable and their levels (significance codes: *0.05, **0.01, ***0.000)

Component	Variable	*z* value	*p* value
1	Depth - deep	1.95	0.051*
2	Depth - deep	3.37	0.001**
	Aspect - east	-8.60	0.000***
	Aspect - south	-10.55	0.000***
	Aspect - west	2.29	0.022*

### Model sensitivity to input parameters

**Fig. 8:**
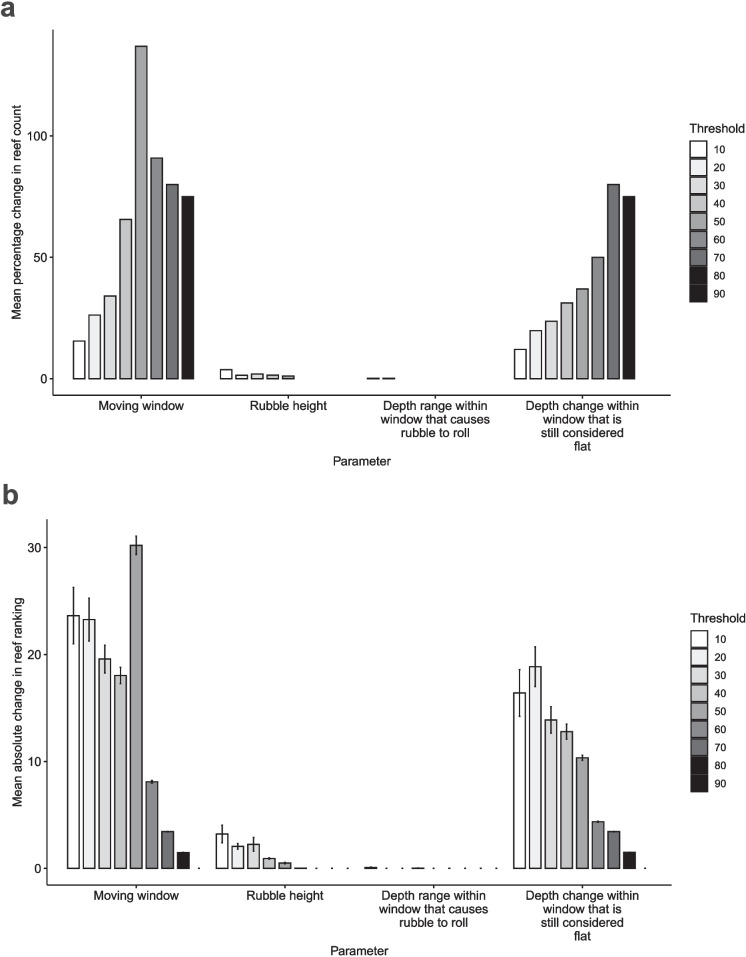
The sensitivity of **a** reef count and **b** reef ranking to the ±20% change in input parameter values. **a** shows the mean percentage change in number of reefs with transects exceeding rubble cover thresholds of 10-90% relative to ± 20% change in parameter values. **b** shows the mean change in ranking of the top 10% reefs across 10–90% rubble cover thresholds relative to ± 20% change in parameter values. The error bars in **b** indicate standard error of the mean ranking change for each threshold

The algorithm was most sensitive to value changes in the *Moving window*, followed by parameters *Depth change within window that is still considered flat*, *Rubble height* and finally *Depth range within window that causes rubble to roll* (Fig. 8). A 20% change in *moving window* resulted in an average change of 58.2 ± 14.4% in the reef count for containing problematic transects and an average absolute change of 14 ± 0.49 in the ranking of the top 10% reefs across threshold categories. In order to provide some perspective, 336 reefs had transects with a rubble cover exceeding 30% in the base case scenario; however, when the *moving window* value was increased by 20%, the reef count decreased to 235. Briggs Reef (No. 1) was initially ranked the highest for having 75% of its transects exceed the 40% rubble cover threshold in the base case scenario, but when the *moving window* value was increased by 20%, only 25% of its transects exceeded the threshold, causing its ranking to drop to fifth place. The reef ranking could fluctuate by a maximum of 17.5% within the range of ranks for all parameters. This implied that few reefs fall outside of the top 10% and that even when the parameters were altered, the algorithm would still place the same reefs as the most vulnerable to rubble accumulation. As shown in Fig. 8, the sensitivity of the model output varied greatly depending on the threshold category. For example, when the threshold category is 50%, a ± 20% change in the value of the parameter, *moving window*, resulted in a 137% change in the number of reefs that are considered problematic. On the other hand, when the threshold category is 90%, the same changes in the parameter value does not affect the number of problematic reefs at all. Although the change in parameter values could result in variable potential rubble cover, the algorithm was consistent in assigning the risk of rubble accumulation by selecting the same top 10% reefs.

## Discussion

Approximately one-quarter of the GBR appears to be unlikely to develop persistent rubble problems, whereas 20% of reefs have the potential to accumulate ~ 30% of rubble across their surface. These reefs can be found throughout the GBR, spanning 13 degrees of latitude. However, rubble-prone reefs are concentrated in the central and southern GBR, which may be attributed to the considerable variation in reef type and geomorphology by latitude (Hopley et al., 2007). The northern region of the GBR has a high density of narrower reefs with steeper reef slopes where rubble may roll off instead of accumulating. Shelf-edge ribbon reefs with steeper slope profiles are limited to the northern region. As the continental shelf gradually widens south of Cairns, reefs are set back from the shelf edge and have gentler slopes, allowing rubble to persist (Hopley et al., 2007).

We found significant effects of aspect in the second group with 31,919 transects, which is consistent with previous studies on how hydrodynamic forcings influence reef geomorphology (Hopley et al., 2007; Montaggioni & Braithwaite, 2009; Scoffin, 1993). Windward, exposed reef slopes are generally steeper, resulting in rubble accumulating as talus at the slope’s foot in deeper waters beyond the depth limits of this study (Montaggioni & Braithwaite, 2009). Reef slopes facing north, or west are typically shielded from the south-easterly trade winds-induced currents that affect the GBR for 9 months of the year, such that they tend to have gentler profiles and accumulate more rubble.

Spur-and-groove systems on reef slopes with high wave exposure, which have been frequently linked to significant rubble accumulation (Duce et al., 2014; Kan et al., 1997; Munk & Sargent, 1954), cannot be differentiated in our analysis due to resolution constraints in the bathymetry and geomorphic data layers. Despite some mapping efforts at the Capricorn Bunker Group (Duce et al., 2014), there is currently insufficient high-resolution data on the spatial distribution of spur and groove structures at a GBR scale. As a result, rubble found in grooves may remain undetected in our analysis, potentially underestimating the reef’s susceptibility to rubble accumulation. Future work could incorporate spur and groove information to enhance the model’s accuracy once it becomes available, allowing researchers to better evaluate the effects of various factors on rubble accumulation.

Despite the uncertainty in current knowledge with which to improve the parameterisation, the approach still serves as a first step in evaluating the vulnerability of the GBR to rubble-induced retardation of coral recovery. The one-dimensional transects were a computationally efficient strategy for sampling reef bathymetric profiles on a large scale, which would otherwise demand a substantial amount of resources. Although the model was moderately sensitive to several input parameters—especially the size of the window used to estimate slope—we found that the ranking of reef susceptibility was pretty robust, with most of the more vulnerable reefs remaining within the top 10%. This is important because it provides grounds to embark on preliminary risk analysis for the GBR and help target large scale validation surveys. In future, the accumulation model can be combined with geospatial data on rubble generation risks and mobilisation rates to develop a comprehensive prediction for reef recovery on a large scale.

One of the greatest limitations of the study is the lack of information on what constitutes a critical cover of rubble to impede coral recovery. Since no study has ever experimentally quantified the critical thresholds for reef recovery, they were chosen based on observed levels on reefs that have exhibited little recovery over 10 years (Mumby, pers. obs.). Although aspect has significant effects on rubble cover in one of the regression groups, it is doubtful whether the magnitude of this effect is sufficient to influence ecological processes. Thus, we conclude that aspect is unlikely to be an important driver of the ability of reefs to accumulate rubble, though it might be important when considering the forces that prevent rubble from stabilising. Indeed, factors including hydrodynamic properties and disturbance regimes have an intricate relationship with rubble dynamics (Harris & Vila-Concejo, 2013; Rasser & Riegl, 2002; Viehman et al., 2018). The study assumed that these factors contribute more to processes of rubble formation and stabilisation than accumulation.

## Conclusions

Coral reefs are experiencing accelerating disturbance (Hughes et al., 2017; McWhorter et al., 2022), and while management can mitigate some types of damage, such as predation from crown-of-thorns starfish (Castro-Sanguino et al., 2023), major events like heatwaves (“coral bleaching”) and cyclones cannot be mitigated directly. Thus, management tends to focus on facilitating the process of coral recovery, whether through improving conditions for coral recruitment and survival (Gove et al., 2023; Mumby et al., 2021) or restoration (Doropoulos & Babcock, 2018). Rubble beds pose a threat to such activities because they can lead to persistent failure of coral recruitment (Dollar & Tribble, 1993; Fox et al., 2003; Riegl, 2001). While we cannot quantify the absolute risk of developing problematic rubble without additional data on rubble stabilisation rates, wave forcing and probabilities of rubble formation, our analysis does suggest that up to 20% of GBR reefs have the sort of bathymetric profile that might trap rubble and allow substantial levels to accumulate. Some areas of the GBR appear to be particularly vulnerable to this issue, including the Pompey Complex and the Capricorn and Bunker Group in the southern GBR. By highlighting that such an important proportion of reefs have the potential to develop problematic rubble, we hope that further research will allow risks to be refined such that managers and reef users can better target where rubble stabilisation might be targeted in future.

### Supplementary information

ESM 1 Flowchart summarising the workflow for mapping reef susceptibility to rubble accumulation (EPS 3457 kb)

## Data Availability

The datasets analysed during the current study can be found online and are publicly available (see Site description and sources of data). The datasets generated during the current study are available from the corresponding author on reasonable request.

## Declarations

All authors have read, understood and have complied as applicable with the statement on “Ethical responsibilities of Authors” as found in the Instructions for Authors.
